# Traditional Chinese Medicine in Patients With Primary Sjogren's Syndrome: A Randomized, Double-Blind, Placebo-Controlled Clinical Trial

**DOI:** 10.3389/fmed.2021.744194

**Published:** 2021-09-28

**Authors:** Huang-Hsi Chen, Jung-Nien Lai, Min-Chien Yu, Chia-Yin Chen, Yi-Ting Hsieh, Yi-Fan Hsu, James Cheng-Chung Wei

**Affiliations:** ^1^Institute of Medicine, Chung Shan Medical University, Taichung, Taiwan; ^2^Division of Allergy, Immunology and Rheumatology, Chung Shan Medical University Hospital, Taichung, Taiwan; ^3^Chinese Medicine Clinical Trial Center, Chung Shan Medical University Hospital, Taichung, Taiwan; ^4^Department of Chinese Medicine, China Medical University Hospital, Taichung, Taiwan; ^5^School of Chinese Medicine, College of Chinese Medicine, China Medical University, Taichung, Taiwan; ^6^School of Post Baccalaureate Chinese Medicine, Tzu Chi University, Hualien, Taiwan; ^7^Department of Chinese Medicine, Taipei Tzu Chi Hospital, Tzu Chi Medical Foundation, New Taipei City, Taiwan; ^8^Graduate Institute of Integrated Medicine, China Medical University, Taichung, Taiwan

**Keywords:** Sjogren's syndrome, Jia-Wei-Xiao-Yao-San, Gan-Lu-Yin, randomized clinical trial, efficacy, safety

## Abstract

**Background:** Sjogren's syndrome (SS) is a chronic inflammatory autoimmune disease mainly characterized by dryness, fatigue, and pain. Current therapies for SS in Western medicine are limited. The purpose of this clinical study was to explore the efficacy and safety of using a traditional Chinese medicine (TCM) formula on patients with primary SS.

**Methods:** We performed a 12-week, randomized, double-blinded, placebo-controlled clinical trial at Chung Shan Medical University Hospital. We included 42 patients with SS between the ages of 20 and 80 years who met the classification criteria of the American and European Consensus Group (AECG). Patients who had other severe systemic manifestations or diseases were excluded from this trial. After screening, patients were randomly assigned to the TCM treatment group or placebo group (ratio of 2:1). We treated the TCM group with 6 g of Gan-Lu-Yin granules after breakfast and 6 g of Jia-Wei-Xiao-Yao-San combined with 1 g of Suan-Zao-Ren-Tang and 1 g of Ye-Jiao-Teng every night after dinner. Patients in the control group were treated with a placebo with the same appearance and flavor but only one-tenth the dosage of that received by the treatment group. The European League Against Rheumatism Sjogren's Syndrome Patient-Reported Index (ESSPRI) was used as the primary endpoint at week 12. Secondary endpoints were the Sjogren's Syndrome Disease Activity Index (SSDAI), physician global assessment (PGA), visual analogue scale (VAS), Multidimensional Fatigue Inventory, Medical Outcomes Survey Short Form-36, and the Pittsburgh Sleep Quality Score (PSQI). Adverse events were also recorded.

**Results:** Of the 42 randomized patients, 28 patients were assigned to the TCM treatment group and 14 patients were assigned to the controlled group. During the study period, 5 patients withdrew from the TCM group and 7 withdrew from the control group. At week 12, the ESSPRI scores of both groups had improved. The ESSPRI score of the treatment group decreased by 0.62 (95% CI *P* = 0.557) and that of the placebo group decreased by 0.91 (*P* = 0.557). However, no significant difference was observed between the two groups. Sleep duration in the PSQI was −0.61, which exhibited an improvement of more than the −0.21 compared with the placebo group (*P* = 0.914).

**Conclusion:** At week 12, the ESSPRI scores did not reveal that the use of the TCM formula was efficacious for treating patients with Sjogren's syndrome. However, the PSQI scores indicated that this formula could prolong patient sleep duration. We also found that this formula could decrease the blood pressure of patients.

## Introduction

Primary Sjogren's syndrome (pSS) is a chronic inflammatory autoimmune disease characterized by sicca syndrome. More than 80% of patients visiting clinics report symptoms of dryness, fatigue, and pain (1). Overall, 20-40% are potentially affected by a severe manifestation of pSS (2, 3). No effective Western medicine therapy has been discovered that benefits patients. Several biologic agents have been tested in clinical trials, but few clinical results have been reported (1). In Eastern medicine, interventions involving traditional Chinese medicine (TCM) have produced heterogeneous and inconsistent results (4). This could be attributed to the lack of a standardized endpoints and evidence-based knowledge regarding certain TCMs. The classification of patients with pSS based on diagnostic methods in Eastern medicine is also a difficult task. The mechanism of Jia-Wei-Xiao-Yao-San (JWXYS) in autoimmune disease was observed to increase synaptic plasticity and decrease the levels of inflammatory markers by upregulating the expression of hippocampal brain-derived neurotrophic factor (BDNF) (5–7). Suan-Zao-Ren-Tang (SZRT) could regulate the gamma-aminobutyric acid-ergic(GABA) system in plasma which is crucial in the etiology of psychiatric disorders (8). The results of studies conducted on animals have indicated that Gan-Lu-Yin (GLY) can influence vascular smooth muscle activity (9) and exert anti-inflammatory effect (10–12).

According to one study, the prevalence rate of Sjogren's syndrome is approximately 0.8% in Taiwan, and the majority of patients are women at a ratio of 3:1. Another study reported that more than 90% of patients with pSS in Taiwan are treated by TCM doctors (13). However, the effectiveness of TCM for patients with pSS in a clinical setting is unknown. Therefore, the purpose of this study was to observe the efficacy and safety of using a frequently prescribed TCM formula selected from records obtained from the National Health Insurance system in Taiwan.

## Methods

### Patient Selection

Patients enrolled in this trial were between the ages of 20 and 80 years, met the classification criteria of the American European Consensus Group, had a European League Against Rheumatism (EULAR) Primary Sjogren's Syndrome Patient-Reported Index (ESSPRI) score greater than three points, and signed an informed consent form after reading and agreeing with the details of this trial. Enrolled patients were required to satisfy either 4 of the 6 of the American European Consensus Group criteria, including No.4 (Histopathology) or No.6 (Autoantibodies), or 3 of the 4 objective criteria (including No.3, No.4, No.5, or No.6). The exclusion criteria were a history of major cardiovascular, pulmonary, or neuropsychiatric disorders, abnormal renal function, low serum white blood cell count, and pregnancy. During the investigation, patients could withdraw from the trial without providing a reason. Patients with a compliance rate of <70%, an ESSPRI score that increased more than 50% twice, or severe adverse events were required to withdraw from the study after approval from the principal investigator.

### Study Design

This was a 12-week, double-blinded, randomized, and placebo-controlled clinical trial. Participants were randomly assigned to either a placebo group or treatment group (ratio of 1:2). Participants were required to return to the clinic for assessments in week 0, 4, 8, and 12.

### Intervention

Patients assigned to the treatment group at the baseline orally received 6 g of Gan-Lu-Yin (GLY) per day after breakfast, and 6 g of Jia-Wei-Xiao-Yao-San (JWXYS) combined with 1 g of Suan-Zao-Ren-Tang (SZRT) and 1 g of Ye-Jiao-Teng (YJT) after dinner every day for 12 weeks. A placebo with one-tenth the dose of the TCM formula and the same appearance was orally administered to patients in the control group every day. Concentrated herbal medicines were manufactured by Chuang Song-Zong pharmaceutical factory, and all met Good Manufacturing Practice requirements. Participants were not permitted to take other traditional Chinese herbal medicines during the study. However, if patients had begun taking the following permitted medications more than 2 weeks before the screening, they were required to remain at a stable dose throughout the 12-week study period: cholinergic medications (Salagen or Exozac), immune modulators (Plaquenil, methotrexate, or boilogics), hypnotics, and anxiolytics (Stilnox, Antivan, or Xanaz), antidepressants, non-steroidal anti-inflammatory drugs, and analgesics (tramadol, Ultracet, or Panadol). Only for safety purposes could the dosage be changed, and such modification was made by the investigator. The Institutional Review Board of Chung Shan Medical University Hospital approved all procedures of this study.

### Endpoints

The primary endpoint was the ESSPRI, which was used to evaluate primary clinical features using a single 0-10 numerical score for each of the three scales (dryness, fatigue, and pain). A higher score indicated more severe symptoms.

Secondary endpoints during the 12-week study period were the EULAR Sjogren's Syndrome Diseases Activity Index, patient global assessment, visual analogue scale, Medical Outcomes Survey Short Form-36, Pittsburgh Sleep Quality Index (PSQI), the Multidimensional Fatigue Inventory, and the Chinese Medical Constitution Questionnaire. The EULAR Sjogren's Syndrome Diseases Activity Index was designed to assess the systemic disease activity in clinical trials for pSS. It reviews 12 selected domains of organ-specific disease activity, each of which is divided into low-activity, moderate-activity, and high-activity levels (2, 14). The Traditional Chinese Medical Constitutional Scale is a tool for diagnosing syndromes and diseases, and its results can be used to determine an appropriate treatment method.

### Safety

Throughout the entire study period, vital signs were recorded and physical examinations were performed at every visit. Laboratory results for serum and biochemistry tests were monitored at weeks 0 and 12. All adverse events were recorded. TCM questionnaires were assessed at weeks 4, 8, and 12. Studying nurses examined patients using a concomitant medication and drug accountability assessment and medication check at each visit.

### Statistical Analysis

Original data were collected and verified by monitors at every visit. Rough data were then input into the database and analyzed by researchers using SAS software. To avoid bias resulting from missing data, the results were modified according to the intention-to-treat principle. Continuous variables of the endpoints were compared with those at the baseline using an analysis of variance or Student's *t*-test. After processing the categorical variables, the researchers used Fisher's exact test, the chi-square test, or the Cochran–Mantel–Haenszel test to analyze the results.

The estimated sample size was calculated under the hypothesis of a 5% type 1 error rate and 20% type 2 error rate. The expected mean changes in ESSPRI scores at week 12 of the intervention was four points in the treatment group and two points in the placebo group, and the standard deviation was set at 2.75. A sample size of 30 patients was assigned to provide 20% power for comparing the outcomes of the two groups using a two-sided *t*-test and Wilcoxon rank-sum test. Because the expected withdrawal rate was 30% and the placebo and TCM groups had a participant ratio of 1:2, 72 observation patients were required prior to beginning enrollment. As a result of budget limitations, recruitment for this study was terminated and analysis was conducted after the enrollment of 42 patients.

## Results

**Figure 1:**
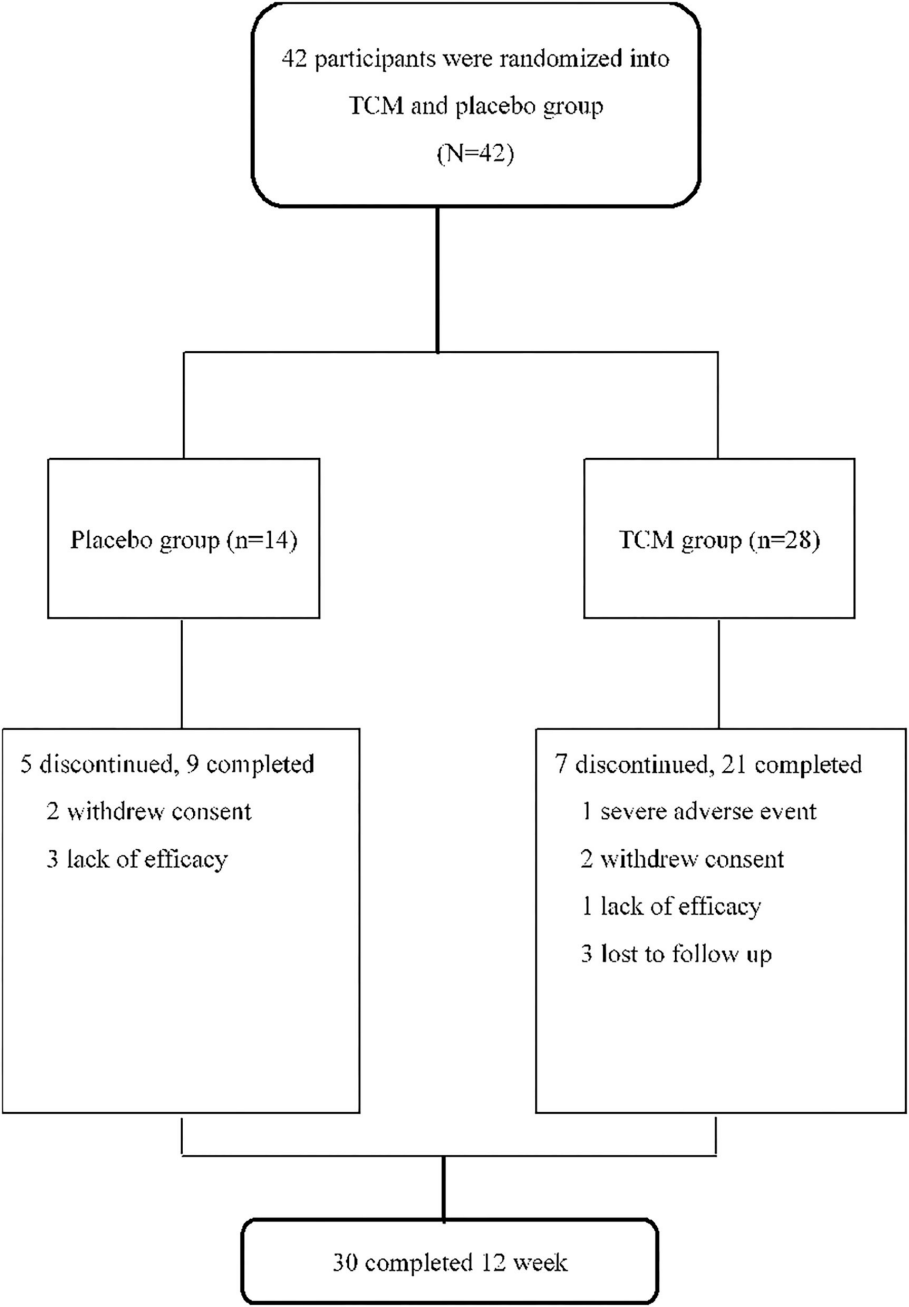
Trial profile. 42 patients were randomized into TCM and placebo group in a ratio of 2:1. In the TCM group, 28 patients received 6 grams of Gan Lu Yin after breakfast, and 6 g of Jia Wei Xiao Yao San combined with 1 g of Suan Zao Ren and 1 g of Ye Jiao Teng after dinner in the evening every day. 14 patients orally received placebo with one-tenth dose of TCM's formula and the same appearance every day.

Of the 42 screened patients, 28 were randomly assigned to the treatment group and 14 were assigned to the placebo group. A total of five participants in the placebo group were withdrawn from the trial; three were withdrawn as a result of inefficient treatment and the other two withdrew voluntarily. In the TCM group, 7 patients voluntarily withdrew and 21 patients completed the 12-week trial. One had a severe adverse event (hydronephrosis), three voluntarily withdrew, one lacked efficacy, and the other three were lost at follow-up (Figure 1). Generally speaking, the health status of patients in the TCM group was worse than that of patients in the placebo group at the baseline. The PSQI scores of patients in the TCM group were higher than those of patients in the placebo group (11.25 ± 4.27 vs. 10.57 ± 5.03; *P* < 0.650). For the Traditional Chinese Medical Constitutional Scale results, all three constitutional types were dominant in the TCM and placebo groups (Table 1). The prevalence of this constitutional type in the control group was higher than that in the TCM group (64.29 vs. 57.14%).

**Table 1:** Baseline characteristics of the study participants.

	**Placebo**	**TCM**	***P*** **-value**
	**(*n* = 14)**	**(*n* = 28)**	
Age	60.14 ± 10.44	58.36 ± 10.48	0.605
Gender	0.333
Women	13(92.86%)	28(100%)	
Height (cm)	156.40 ± 6.25	157.20 ± 5.74	0.687
Weight (kg)	58.79 ± 6.82	53.21 ± 8.08	0.033^*^
BMI	23.98 ± 2.08	21.52 ± 2.96	0.008^*^
SBP (mmHg)	120.90 ± 13.81	120.60 ± 14.32	0.945
DBP (mmHg)	73.71 ± 8.96	72.64 ± 9.39	0.725
MBP (mmHg)	89.45 ± 9.83	88.63 ± 10.08	0.803
**Biochemistry**			
ESR(mm/h)	18.5(15)	24(22.5)	0.225
Hs-CRP(mg/dl)	0.04(0.2)	0.05(0.08)	0.592
GPT(IU/L)	21.5(7)	19(7)	0.676
CK (IU/L)	93(63)	83.5(48.5)	0.92
Creatinine (mg/dl)	0.72(0.19)	0.71(0.2)	0.65
RF(IU/ml)	20(0)	20(6.7)	0.432
C3(mg/dl)	96.15 ± 12.97	92.84 ± 16.84	0.523
C4(mg/dl)	20.94 ± 3.62	18.52 ± 4.15	0.071
IgG (mg/dl)	1,370(300)	1,505(485)	0.303
IgA(mg/dl)	297 ± 159.6	282.3 ± 107.2	0.726
anti-SSA	0.832		
Negative	6(42.86%)	13(46.43%)	
Positive	8(57.14%)	15(53.57%)	
anti-SSB	0.619		
Negative	10(71.43%)	22(78.57%)	
Positive	4(28.57%)	6(21.43%)	
ESSPRI	5.98 ± 1.12	6.24 ± 1.31	0.528
Severity of dryness during the last 2 weeks	6.50 ± 1.79	7.11 ± 1.40	0.234
Severity of fatigue during the last 2 weeks	6.07 ± 1.82	6.36 ± 1.89	0.643
Severity of pain during the last 2 weeks	5.36 ± 1.98	5.25 ± 2.22	0.88
(Joint or muscular pains in Arms or legs)			
ESSDAI	0.00(2.00)	0.00(1.00)	0.703
PGA	5.95(2.00)	6.00(2.00)	0.52
VAS	5.00(3.00)	5.75(3.50)	0.735
PSQI Score	10.57 ± 5.03	11.25 ± 4.27	0.65
**SF36**			
Physical functioning	82.5(25)	67.5(30)	0.208
Role-physical	87.5(100)	25(100)	0.142
Bodily pain	62(18)	62(11)	0.148
General health	45(20)	32.5(27.5)	0.055
Vitality	52.86 ± 20.64	42.32 ± 17.87	0.1
Social functioning	87.5(37.5)	75(31.25)	0.29
Role-emotional	50(100)	66.67(83.33)	0.569
Mental health	56 ± 24.66	52 ± 20.57	0.581

The meaning of bold values indicated that ^*^P < 0.05, ^**^P < 0.06. The meaning of

* indicated that ^*^P < 0.05, ^**^P < 0.06.

**Figure 2:**
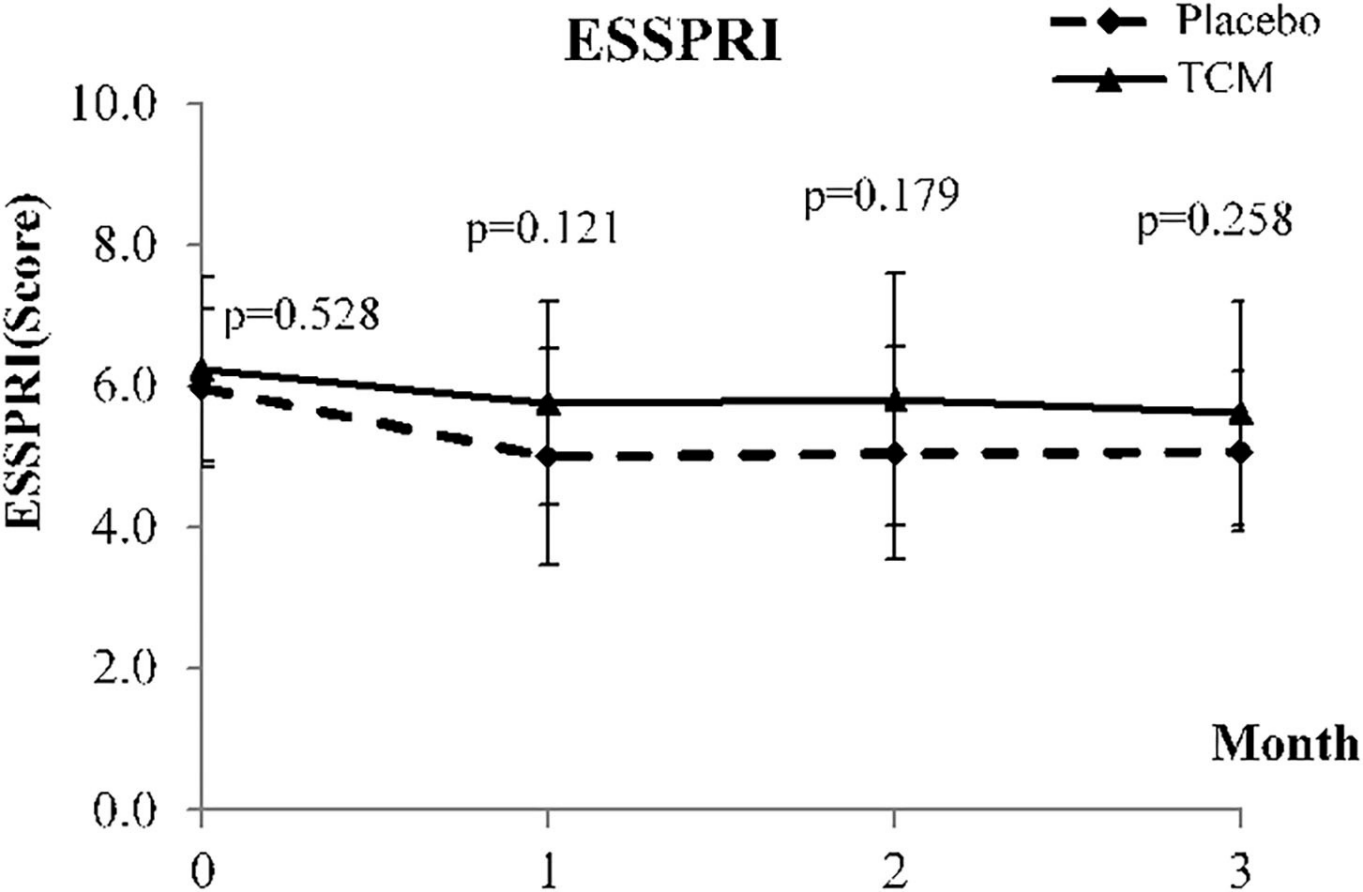
Means and standard deviaion of ESSPRI at baseline and weeks 4, 8, and 12.

**Figure 3:**
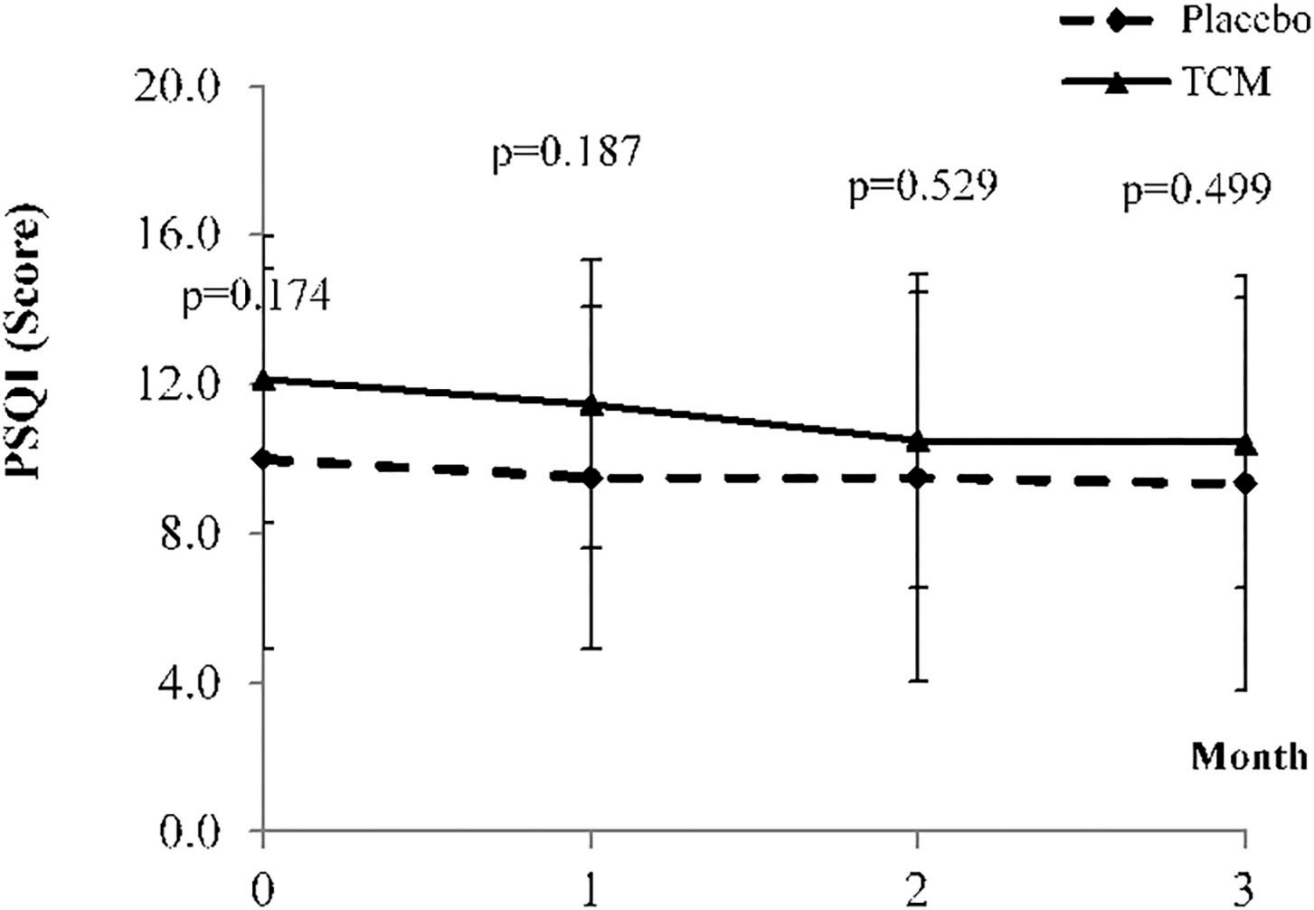
Means and standard deviation of PSQI at baseline and weeks 4, 8, and 12.

At week 12, although there are no significant change in most basic and biochemistry data, the IgG level of the treatment group had decreased by 39.64 (*P* = 0.042^*^), significantly. The ESSPRI scores of both groups had improved. However, the ESSPRI scores of the treatment group decreased by 0.62 (*P* = 0.557) and those of the placebo group decreased by 0.91 (*P* = 0.557). The ESSPRI scores of patients in the treatment group were higher than those of patients in the control group (Table 2; Figure 2). Compared to week 0, the ESSDAI scores of the treatment group significantly improved. But no significant difference in the ESSDAI scores was found between the two groups. Moreover, the PGA scores had significantly decreased in the treatment group (−1.37 ± 2.37^*^, *P* = 0.075) than in the placebo group (−0.24 ± 1.36, *P* = 0.075). In the facet of General Health in SF-36, the scores of the treatment group (7.14 ± 11.82^*^, *P* = 0.330) had obviously raised than those of the placebo group (−3.57 ± 9.29, *P* = 0.330). Sleep duration for the PSQI was −0.61, exhibiting an improvement of more than the −0.21 compared with the placebo group (*P* = 0.914; Figure 3). None of the other secondary endpoints for the two groups demonstrated that the TCM formula was efficacious. One severe adverse event was caused by hydronephrosis, but this was unrelated to the TCM formula.

**Table 2:** Changes in primary and secondary endpoints at week 12.

	**Placebo (*n* = 14)**	**TCM (*n* = 28)**	***P*** **-value**
Weight (kg)	−0.29 ± 2.09	0 ± 1.41	0.603
SBP (mmHg)	−1.14 ± 10.43	3 ± 20.21	0.386
DBP (mmHg)	−3.86 ± 11.55	−1.5 ± 7.46	0.428
MBP (mmHg)	−2.95 ± 9.42	0 ± 8.56	0.314
**Biochemistry**			
ESR(mm/h)	1.07 ± 3.93	−0.39 ± 5.72	0.395
Hs-CRP(mg/dl)	−0.01 ± 0.05	0.02 ± 0.15	0.429
GPT(IU/L)	1.29 ± 5.41	−2.36 ± 10.36	0.142
CK (IU/L)	−6.43 ± 32.43	−4.57 ± 48.54	0.898
Creatinine (mg/dl)	0.02 ± 0.08	0 ± 0.08	0.402
RF(IU/ml)	−4.07 ±−13.03	5.53 ±−10.37	0.282
C3(mg/dl)	−0.91 ± 4.84	−2.17 ± 7.31	0.564
C4(mg/dl)	−1 ± 1.94	5.16 ± 28.91	0.271
IgG (mg/dl)	42.86 ± 99.03	−39.64 ± 128.9	0.042^*^
IgA(mg/dl)	−5.76 ± 21.96	−7.96 ± 25.46	0.784
anti-SSA	1.000		
Negative	6(42.86%)	12(42.86%)	
Positive	8(57.14%)	16(57.14%)	
anti-SSB	0.459		
Negative	9(64.29%)	22(78.57%)	
Positive	5(35.71%)	6(21.43%)	
ESSPRI	−0.91 ± 1.26*	−0.62 ± 1.57	0.557
Severity of dryness during the last 2 weeks	−0.93 ± 1.69	−1.14 ± 1.74	0.706
Severity of fatigue during the last 2 weeks	−0.93 ± 2.5	−0.75 ± 2.68	0.836
Severity of pain during the last 2 weeks (Joint or muscular pains in Arms or legs)	−0.86 ± 1.88	−0.07 ± 2.09	0.242
ESSDAI	−0.71 ± 1.27	–0.57 ± 1.07^*^	0.703
PGA	−0.24 ± 1.36	–1.37 ± 2.37^*^	0.057
VAS	−0.47 ± 2.27	−0.82 ± 2.38	0.654
PSQI Score	−0.71 ± 1.86	−1.36 ± 3.63	0.453
**SF36**			
Physical functioning	2.86 ± 9.35	−0.36 ± 15.27	0.476
Role-physical	10.71 ± 28.95	7.14 ± 40.74	0.772
Bodily pain	4.57 ± 10.43	4.68 ± 19.89	0.982
General health	3.57 ± 9.29	7.14 ± 11.82^*^	0.330
Vitality	7.86 ± 18.37	0.54 ± 15.17	0.177
Social functioning	1.79 ± 21.29	5.36 ± 15.38	0.537
Role-emotional	4.76 ± 31.64	−1.19 ± 33.31	0.582
Mental health	6.57 ± 11.16^*^	2.71 ± 14.49	0.388

The meaning of bold values indicated that ^*^P < 0.05, ^**^P < 0.06. The meaning of

* indicated that ^*^P < 0.05, ^**^P < 0.06.

## Discussion

In this 12-week, double-blinded, placebo-controlled clinical trial, we concluded that GLY and JWXYS TCM formula had no significant effect on patients with pSS compared with a placebo, according to ESSPRI scores. Worthy of note, compared with the placebo, the TCM formula could prolong total sleep time by approximately half an hour, according to the PSQI scores. And, IgG which was a well known predisposition of pSS to immunological hyper-responsiveness decreased significantly at the end of the study. The decrease of ESSDAI and PGA and the improvement of General Health were observed after receiving TCM treatment. Safety profiles revealed that this formula could be tolerated by patients with pSS, although frequent mild adverse events in the gastrointestinal system were reported.

The poor clinical performance of TCM could be attributed to several factors in this trial. First, the TCM formula or dosage we used to treat patients with pSS may not have been applicable to the population we included. Although the clinical manifestations of the TCM group were more severe at the baseline, there were not statistically different between two groups. The clinical manifestations of the TCM group were more severe at the baseline. Applying other herbal medicines or dosages as interventions would have been more beneficial for patients. Second, the follow-up period was too short to demonstrate the efficacy of herbal medicine. Evidence-based data regarding GLY and JWXYS are still lacking, and most TCM physicians prescribe treatment according to their clinical experience without strong support from scientific evidence. Third, the health statuses of patients in the two groups were not equivalent at the baseline. Unbalanced variables may have led to deviations in the results. Enlargement of the sample size could reduce this deviation.

The improvement in sleep quality and duration could be a result of the JWXYS and SZRT. Previous evidence-based research demonstrated that a JWXYS and SZRT formula could ameliorate sleep-related problems in climacteric women. JWXYS is effective for treating insomnia, and SZRT can prolong sleep duration by ~1 h after 1 month of treatment (15–17). The results of studies conducted on animals have indicated that GLY can influence vascular smooth muscle activity (9).

This study had some limitations. First, the sample size is relatively small and study duration is short. But for a pilot study, we believed that this exploratory study can still provide important information. Second, the dosage and herbal combinations were decided by clinical experience of TCM experts, but not pre-clinical study nor phase II dose-ranging studies. However, we believe that TCM clinical trials can be started by phase II pilot study, since these formulas had been used in clinical practice for hundreds of years. Finally, the study results cannot be generalized to other population, other TCM formula or dosage because this is a single-center, small scaled exploratory study (18–23).

## Conclusion

At week 12, the ESSPRI scores did not indicate the efficacy of the TCM formula in treating patients with Sjogren's syndrome. However, the PSQI scores revealed that this formula could prolong the sleep duration of patients. We also discovered that this formula could decrease the serum level of IgG and blood pressure at the end of the study. Only few mild adverse events were observed.

## Data Availability Statement

The original contributions presented in the study are included in the article/Supplementary Material, further inquiries can be directed to the corresponding author/s.

## Ethics Statement

Ethical review and approval was not required for the study on human participants in accordance with the local legislation and institutional requirements. The patients/participants provided their written informed consent to participate in this study.

## Author Contributions

H-HC, J-NL, M-CY, and C-YC contributed to conception and design of the study. H-HC wrote the first draft of the manuscript. J-NL contributed to funding acquisition and project administration. M-CY and C-YC organized the database and performed the statistical analysis. Y-TH and Y-FH wrote sections of the manuscript. JW contributed to the design of the study, conducting of the experiments, interpretation of the data and critical revision/final approval of the manuscript. All authors contributed to manuscript revision, read, and approved the submitted version.

## Funding

This study was supported in part by Taiwan Ministry of Health and Welfare Clinical Trial Center, Grant Number: MOHW-110-CMC-03.

## Conflict of Interest

The authors declare that the research was conducted in the absence of any commercial or financial relationships that could be construed as a potential conflict of interest.

## Publisher's Note

All claims expressed in this article are solely those of the authors and do not necessarily represent those of their affiliated organizations, or those of the publisher, the editors and the reviewers. Any product that may be evaluated in this article, or claim that may be made by its manufacturer, is not guaranteed or endorsed by the publisher.

## Abbreviations

SS: Sjogren's syndrome
TCM: Traditional Chinese Medicine
AECG: American and European Consensus Group
ESSPRI: The European League Against Rheumatism Sjogren's Syndrome. Patient-Reported Index
SSDAI: Sjogren's Syndrome Disease Activity Index
PGA: Physician Global Assessment
VAS: Visual Analogue Scale
PSQI: Pittsburgh Sleep Quality Score
EULAR: European League Against Rheumatism
GLY: Gan-Lu-Yin
JWXYS: Jia-Wei-Xiao-Yao-San
SZRT: Suan-Zao-Ren-Tang
YJT: Ye-Jiao-Teng.
